# Evaluation of the Accuracy, Surgical Time, and Learning Curve of Freehand, Static, and Dynamic Computer‐Assisted Implant Surgery in an In Vitro Study

**DOI:** 10.1111/clr.14403

**Published:** 2025-01-21

**Authors:** Joscha Gabriel Werny, Shengchi Fan, Leonardo Diaz, Bilal Al‐Nawas, Keyvan Sagheb, Matthias Gielisch, Eik Schiegnitz

**Affiliations:** ^1^ Department of Oral and Maxillofacial Surgery—Plastic Operations University Medical Center Mainz Mainz Germany; ^2^ Oral Surgery and Implantology, Faculty of Medicine and Health Sciences University of Barcelona Barcelona Spain; ^3^ Postgraduate School, Faculty of Dentistry Universidad de Chile Santiago Chile

**Keywords:** computer‐aided implant surgery, implant accuracy, learning curve, surgeon‐reported outcome, surgical time

## Abstract

**Objectives:**

This experimental study compared the accuracy of implant insertion using the free‐hand (FH) technique, static computer‐aided surgery (S‐CAIS), or dynamic computer‐assisted surgery (D‐CAIS) and to evaluate the correlation of learning curves between surgeons' experience and surgical time.

**Materials and Methods:**

Thirty‐six models were randomly assigned to three groups (FH, *n* = 12; S‐CAIS, *n* = 12; D‐CAIS, *n* = 12). Each model was planned to receive four implants in the maxillary anterior and posterior regions. Twelve participants, six experienced surgeons, and six dental students were included in this study. The primary outcome was the deviation between the planned and final implant placement from each group. Secondary outcomes were each technique's learning curve regarding surgical time.

**Results:**

The average deviation at implant platform, apex and gradual deviation with FH technique were 1.31 ± 0.88 mm, 1.75 ± 0.9 mm and 6.67° ± 3.70°, respectively. The average deviation of implant platform, apex and angular in S‐CAIS were 0.67 ± 0.32 mm, 1.00 ± 0.39 and 2.66° ± 1.77°, respectively. The average deviation of implant platform, apex and angular in D‐CAIS were 1.14 ± 0.70 mm, 1.23 ± 0.58 and 3.20° ± 2.16°, respectively. Significant discrepancies at the implant platform, implant apex, and angular deviation were found between all surgical methods (*p* < 0.016). Learning curves were evident after multiple implant insertions using both freehand and S‐CAIS.

**Conclusion:**

The findings indicate that computer‐assisted implant insertion leads to a more precise implant alignment than implants inserted freehand in an experimental set‐up.

## 1 Introduction

The precise placement of dental implants is essential to the long‐term prognosis, marginal bone stability, and the prevention of soft tissue complications. With precise planning, surgeons can avoid damaging critical anatomical structures such as major blood vessels, thin bone structures, and nerves. Furthermore, the precise placement of dental implants positively affects the success of implant‐supported dentures (Pozzi et al. 2021) and the esthetic outcomes (Forna and Agop‐Forna 2019). With the help of computer‐assisted implant surgery (CAIS), surgeons can reproduce the planned implant position during the surgery with clinically adequate accuracy (Gargallo‐Albiol et al. 2019; Hämmerle et al. 2015).

Nowadays, CAIS can be performed with a three‐dimensional (3D) printed static template with guiding holes or a surgical navigation system with a tracking camera. Three different types of static computer‐aided surgery (S‐CAIS) have been introduced. These distinguish by each other by the number of guided drills; just the pilot drill, several drill sizes or even including the placement of the implant. Studies have proven that each type of S‐CAIS in different sceneries with teeth‐supported guidance could generally achieve the highest accuracy with fully guided surgery (Gourdache et al. 2024).

Compared to the S‐CAIS, the advantages of the dynamic computer‐assisted surgery (D‐CAIS) are the real‐time visualization of the planned and actual position of the osteotomy during the surgery. The greater flexibility of D‐CAIS allows it to adjust the implant position during surgery and enables it to achieve the optimal implant position in different situations. Thus, the surgeon can prepare the osteotomy and avoid injuring critical anatomical structures from the benefit of real‐time navigation monitoring. Changing the clinical view from the surgical site to the monitor could be challenging because the surgeon needs to adapt to multiple real‐time planes for implant osteotomy with a strong sense of orientation.

Despite the surgical skill and experience requirement, a comprehensive understanding of D‐CAIS workflow and the navigation approach are vitally important for precise results. Three fundamental steps of D‐CAS are registration, calibration, and tracking, which together enable the real‐time mapping of the instruments to the patient's pre‐operative planning (Soteriou et al. 2016; Widmann, Stoffner, and Bale 2009; Wu et al. 2019). The markers must be clearly identified and rigidly fixed to reduce the registration error. During the surgical procedure, the marker must be identified by the navigation tool, allowing the camera to provide the real‐time localization of the handpiece and the surgical field. The stability and distribution of registration markers are the main factors for the accuracy of the navigation. With this setup, D‐CAIS has been reported to have a longer surgical time than free‐hand (FH) implant placement (Jorba‐García et al. 2019).

After repeated use, an actual learning curve regarding the accuracy of placed implants was observed with surgical navigation (Block et al. 2017; Feng, Yao, and Yang 2022; Marques‐Guasch et al. 2022). This workflow appears to bring advantages for patients and clinicians, especially during the training of novice surgeons (D'Haese et al. 2017; Kunakornsawat et al. 2023). However, evaluating the real CAIS learning curve in an in vivo study without interfering factors, such as bleeding, mouth opening, and surgeon's experience and fatigue, is difficult.

The present study compared three approaches of preparing the osteotomy supported by FH, S‐CAIS, or D‐CAIS technique for implant placement in a 3D‐printed model. The primary outcome was the accuracy of placed implants compared to the preoperatively planned position. The secondary outcome was to evaluate the correlation of the learning curve regarding surgical time, surgeon's experience, and surgeon‐reported outcomes.

## 2 Material and Methods

### 2.1 Scanning Procedures

A dental model with missing teeth 16, 11, and 25–27 in the maxilla was printed by a filament printer (Raise3D E2, Raise3D, Netherlands) using a biopolymer filament (GreenTEC Pro, Extrudr, Austria). The printing parameters were set to simulate bone structure: the cortical thickness of 1.2 mm and an infill of less than 50% with a honeycomb structure. Digitization of the model was performed with double scans technique by using an intra‐oral scanner (IOS) (TRIOS move+, 3Shape, Denmark) and cone‐beam computed tomography (CBCT) (Accuitomo 170, J. Morita Inc., Kyoto, Japan, 2 mA, 63 kV, 0.16 × 0.16 × 0.16 mm voxel size, and 16 × 8 cm field of view) to generate standard triangle language (STL) and digital imaging and communications in medicine (DICOM) data of the model.

### 2.2 Digital Planning Procedure

The DICOM data generated by the CBCT scan and STL data of the IOS were imported and merged in the digital planning software (coDiagnostiX, Dental Wings GmbH, Germany). Digital wax‐up in edentulous gaps was performed on the 3D model. Four Implants (three Straumann SP/RT TLX Ø 3.75 × 12 mm and one Straumann RC BLX Ø 3.75 × 10 mm) virtual position were prosthetically driven planned with sufficient bone surrounding. TLX implants were placed in regions 16, 24, and 26, while BLX implants were placed in region 11. Three surgeons (J.G.W., L.D., and S.F.) performed digital planning of the implant positions. Navigation planning was then transferred to the burr‐software (DENACAM, mininavident AG, Switzerland). Surgical guides were printed by a digital light processing 3D printer (SolFlex 170, VOCO, GmbH, Germany) using a light‐curing resin for generative production of dental surgical guides (V‐Print SG, VOCO GmbH, Germany) for static and dynamic navigation. The offset parameter of the surgical guide between the IOS and the template was 0.15 mm, enabling a stable retentive connection with the dentition and reducing the risk of template fracture. The thickness of the template was 2.7 mm, and sleeves (T‐sleeve, Straumann, Switzerland) were added to complete the guided system.

### 2.3 Surgical Implant Placement

A total of 12 participants were included in this study, divided into 2 two teams regarding the clinical experience. The first team consisted of experienced surgeons (B.A., K.S., E.S., L.D., S.F., M.G.) who have placed more than 100 implants per year. The second team consisted of dental students (GK, VB, LW, EK, AS, and JW) with limited clinical experience in implant surgery. Thirty six models with numbers marked (1–36) were randomly assigned to FH (Group A), S‐CAIS (Group B), and D‐CAIS (Group C). Every participant received three models with a randomized number above. Afterward, three surgical techniques were applied for four implant placements in each model. According to the study protocol, the order of implant placement was 16, 11, 24, and 26, respectively. Implant insertion in the first model was performed FH (Group A), the second with S‐CAIS (Group B), and the third with D‐CAIS (Group C). The surgical guidance used in Group B was designed fully guided, while all surgical techniques were performed with the surgical kit (VeloDrill, Straumann, Switzerland), following the manufacturer's recommendations. For the implant bed preparation, the speed of the drill rotation (1200 rpm) and manual insertion were performed in all techniques. To simulate clinical scenery, the models were screw‐retained (M6, Bauhaus, Germany) in a phantom head (Frasaco AG‐3, Frasaco, Germany). During the surgeries, the time for each implant insertion was measured from the beginning of the surgery to the end of the final alignment.

#### 2.3.1 FH (Group A) Protocol

The drilling protocol generated by the planning software gave instructions for the planned implant position. The protocol included three‐dimensional images of the intentional implant position in multiple planes and the correct order of drills for the osteotomy. Additionally, the crestal distances of the implant to the adjacent teeth were given in mm.

#### 2.3.2 S‐CAIS (Group B) Workflow

**FIGURE 1:**
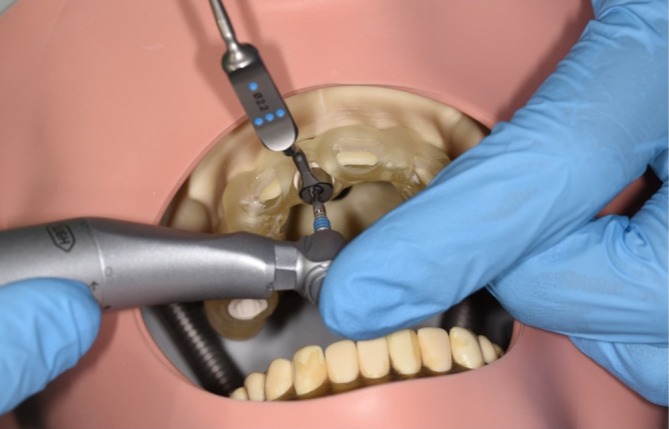
Implant bed preparation with static CAIS.

Each participant was provided with a fully guided surgical template. Drilling was performed with the same surgical guide for all participants. The drilling protocol included instructions of the order and length of drills. Spoons (VeloDrill, Straumann, Switzerland) were necessary to fit the diameter of the sleeve and each drill (Figure 1). Following the drilling procedure, the implants were inserted manually by the guidance of a spoon.

#### 2.3.3 D‐CAIS (Group C) Workflow

The D‐CAIS‐assisted implant placement was performed according to the instructions of the navigation system manufacturer (Denacam, mininavident AG, Schweiz). The dynamic surgical system uses a stereoscopic optical camera attached to the headpiece. A small prefabricated intraoral marker was attached to the model to coordinate the planned implant position and the real‐time position of the drill during surgery. The surgeon can recognize real‐time deviations in the entry point, apex, and angulation via a navigation monitor. The workflow requires three essential steps to perform navigated implant surgery: planning, tracing, and placement (Fan, Gielisch, et al. 2023).

##### 2.3.3.1 Planning Phase

The planned implant positions were duplicated and imported into the navigation system in group C. Afterward, a supporting structure for the registration marker was designed and printed with a teeth‐supported template in the region of 23, which allowed the camera to track the marker during implantation.

##### 2.3.3.2 Registration, Calibration, Placement

**FIGURE 2:**
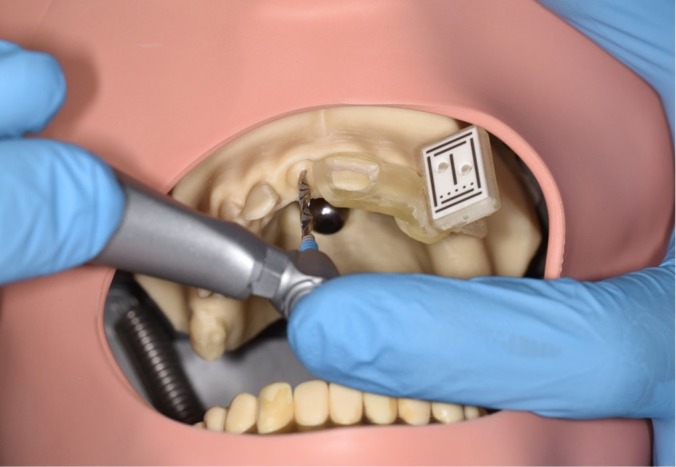
Implant bed preparation with dynamic CAIS.

The tracking marker was firmly attached to the template for the registration process. Before drilling, the drill needed to be calibrated by the calibration tool provided by the manufacturer (Figure 2). In this step, the length and diameter of the drill, as well as the angulation of the handpiece, were able to be measured and transferred to the navigation system automatically. During the osteotomy, real‐time visualization of the drill position of entry point, angular deflection, and drill depth were constantly shown. Each participant performed calibration and implant bed preparation with different drills according to the manufacturer's guidelines.

#### 2.3.4 Surgeon‐Reported Outcome (SRO)

After implant placement, participants were asked to answer an anonymous survey about their experiences using different implant insertion techniques (self‐confidence, spatial freedom, mindfulness, subjective feeling of accuracy, and possibility of repeated use). All questions were asked consecutively and in the same order with a scale of 0–5 to enable a differentiated answer. An online form (Google Forms, Google Inc., USA) was used to collect the participants' answers.

#### 2.3.5 Data Collection and Error Evaluation

**FIGURE 3:**
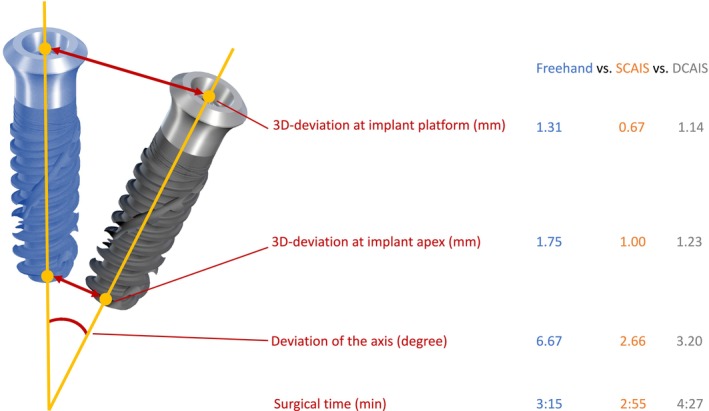
Drafted comparison of the degree's planned and final implant positions, the 3D deviation at platform and apex in mm, and surgical time. Behind the description, average measurements are shown. The blue implant represents the digitally planned position, and the grey implant represents the final implant position. The yellow dots represent the center of the apex and platform, while the line indicates the axis of the implant.

**FIGURE 4:**
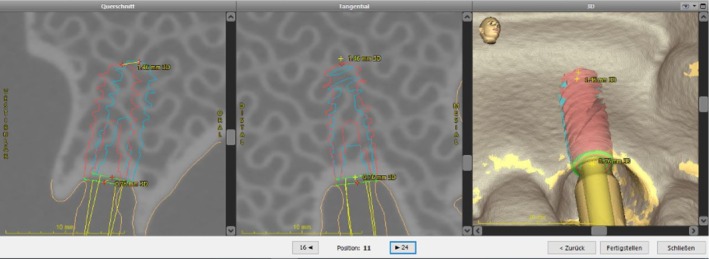
Comparison of the planned and final implant positions. The blue implant is the digital planning, while the red one shows the final position.

**FIGURE 5:**
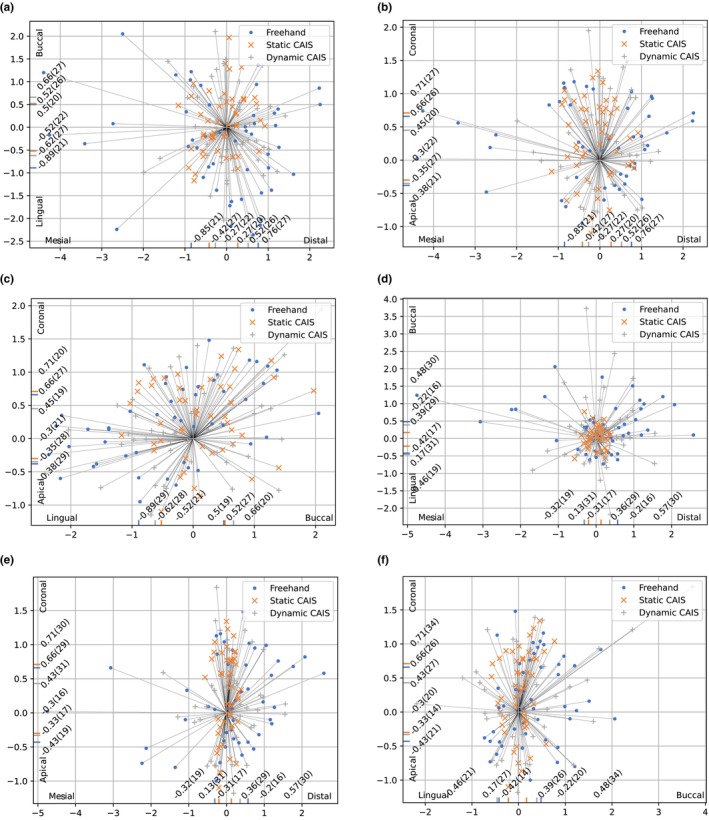
The scatter plot illustrating the direction of deviation for each group. (a) Buccolingual and mesiodistal planes at implant platform. (b) Mesiodistal and apicocoronal planes at implant platform. (c) Buccolingual and apicocoronal planes at implant platform. (d) Buccolingual and mesiodistal planes at implant apex. (e) Mesiodistal and apicocoronal planes at implant apex. (f) Buccolingual and apicocoronal planes at implant apex.

After the placement, scanbodies (CARES, Straumann, Switzerland) were fixed to the implants and then scanned by an IOS (TRIOS 4 move+, 3Shape, Denmark) for accuracy analysis. The placed data was overlaid on the planned data in the planning software. Scanbodies were detected by the implant planning program (coDiagnostiX, Dental Wings GmbH, Germany). Following, the locations of the placed implant were projected into the 3D model at the exact position. Then, the implant locations were compared with the planned positions for each model (Figures 3, 4). Finally, the deviations were automatically calculated in terms of coronal deviations (mm) (Figure 5a–c), apical deviations (mm) (Figure 5d–f), and angular deviations (degree). The coronal and apical deviation measurements included the deviation in mesial‐distal, oral‐vestibular, and apical‐coronal directions. The learning curve was measured with the accuracy and costed time during implant surgery.

### 2.4 Sample Size Calculation

The sample size was calculated based on the means and standard deviations of the 3D deviation at the implant apex of three groups using both CAIS and freehand methods, as reported in a previous study (Block et al. 2017) (1.29 ± 0.65, 1.52 ± 0.78, and 2.27 ± 1.02 mm, respectively). The sample size required for the study comprised 100 implants, as detected by (G*Power version 3.1.9.5) statistical software. The significance level (α) was set at 0.05, with a power (1‐β) of 0.95 and an allocation ratio of 1. The effect size was found to be 0.43. Since 75% of the implants were tissue‐level implants, the sample size was 108 for tissue‐level and 36 bone‐level implants, arranged into three groups of 48.

### 2.5 Statistically Analysis

Statistical analysis was performed using SPSS 27.0 software. The normality of the primary and secondary outcome data distribution was assessed with the Shapiro–Wilk test. Surgical time across different groups, varying surgeon experience and insertion methods, and coronal, apical, and angular deviations, overall and in different implant regions, were compared with a one‐way ANOVA followed by a Scheffe post hoc test. A 2‐way ANOVA was performed considering surgeon experience and the implant insertion method to determine if the differences among the methods remain significant even after adjusting for surgeon experience. *p* < 0.05 were defined as statistically *t*. The questionnaire was also analyzed using SPSS 27.0 software, and learning curves were analyzed by linear mixed models random effects without assumptions of covariances and a Kenward–Roger approximation.

## 3 Results

### 3.1 Deviation at Implant Platform and Apex

The mean deviation at implant platform and implant apex in the FH group was 1.31 ± 0.88 mm (95% CI: 1.06–1.57) and 1.75 ± 0.9 mm (95% CI: 1.48–2.01), the S‐CAIS group was 0.67 ± 0.32 mm (95% CI: 0.58–0.76) and 1.00 ± 0.39 mm (95% CI: 0.89–1.11), and D‐CAIS group was 1.14 ± 0.70 mm (95% CI: 0.94–1.34) and 1.23 ± 0.58 mm (95% CI: 1.06–1.39), respectively. The angular deviation in FH, S‐CAIS, and D‐CAIS groups were 6.67° ± 3.70° (95% CI: 5.60–7.75), 2.66° ± 1.77° (95% CI: 2.14–3.17), and 3.20° ± 2.16° (95% CI: 2.58–3.83), respectively.

Significant differences were found between S‐CAIS and FH in angular deviation (*p* < 0.001), deviation at the implant platform (*p* < 0.001), and apex (*p* < 0.001). Between D‐CAIS and FH, there were significant differences in the angular deviation (*p* < 0.002), implant apex (*p* = 0.043), and surgical time (*p* = 0.016). Additionally, S‐CAIS and D‐CAIS significantly differed in the implant platform deviation (*p* = 0.005) and the surgical time (*p* = 0.002).

**FIGURE 6:**
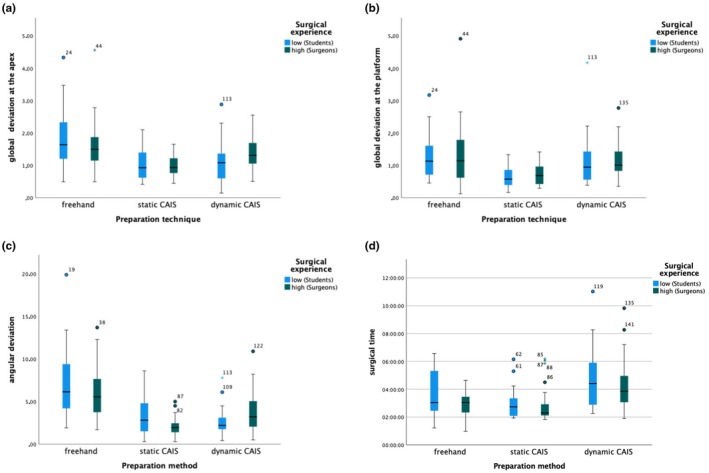
(a–d) Boxplots showing the difference between surgeons with a high level of experience and students with a low level of experience in implant dentistry in terms of implant deviation at apex, platform, angular and surgical time.

The comparison of participants with different skill levels showed that the team of experienced surgeons had significantly reduced surgical time during FH implantation compared to the team of non‐experienced surgeons (*p* < 0.003) (Table 1). S‐CAIS was significantly more accurate in implant angulation when experienced surgeons performed surgeries (*p* = 0.002) (Table 2). No statistically significant differences were found between other parameters or during D‐CAIS between different skilled surgeons (Figure 6a–d, Table 3). Implants placed in the multiple missing teeth gaps with FH (24 and 26) were associated with more deviations at the implant platform than in the single tooth gap (11 and 16). The lower deviation in the multiple missing teeth gaps was observed when using S‐CAIS or D‐CAIS.

**TABLE 1:** Deviations of implant positions and operation times of students and surgeons, using the preparation method freehand.

Skill level	Surgical time (min)	Angular deviation (°)	Deviation at the platform (mm)	Deviation at the apex (mm)
Surgeons	2:54 ± 0.55	6.23 ± 3.08	1.33 ± 1.03	1.65 ± 0.83
Students	3:35 ± 1:37	7.11 ± 4.25	1.29 ± 0.73	1.84 ± 0.98
Significant	*p* = 0.002*	*p* = 0.236	*p* = 0.380	*p* = 0.322

*Note*: *indicates a significant difference (*p* < 0.05).

**TABLE 2:** Deviations of implant positions and operation times of students and surgeons, using the preparation method S‐CAIS.

Skill level	Surgical time (min)	Angular deviation (°)	Deviation at the platform (mm)	Deviation at the apex (mm)
Surgeons	2:53 ± 1:21	2.11 ± 1.11	0.72 ± 0.33	0.98 ± 0.31
Students	2:57 ± 1:04	3.20 ± 2.13	0.62 ± 0.32	1.02 ± 0.46
Significant	*p* = 0.322	*p* = 0.002*	*p* = 0.700	*p* = 0.068

*Note*: *indicates a significant difference (*p* < 0.05).

**TABLE 3:** Deviations of implant positions and operation times of students and surgeons, using the preparation method D‐CAIS.

Skill level	Surgical time (min)	Angular deviation (°)	Deviation at the platform (mm)	Deviation at the apex (mm)
Surgeons	4:12 ± 1:58	3.75 ± 2.46	1.14 ± 0.54	1.36 ± 0.48
Students	4:42 ± 2:11	2.65° ± 1.68°	1.14 ± 0.83	1.09 ± 0.64
Significant	*p* = 0.472	*p* = 0.123	*p* = 0.245	*p* = 0.288

Surgical time for FH, S‐CAIS, and D‐CAIS were 3:15 ± 1:21 min (95% CI: 2:51–3:38), 2:55 ± 1:12 min (95% CI: 2:34–3:16), and 4:27 ± 2:04 min (95% CI: 3:51–5:03) (Table 4).

**TABLE 4:** Variations of implant positions and operation times of all participants, divided according to the preparation method.

	Freehand		S‐CAIS		D‐CAIS		*p*	*p*	*p*	*p*
	Mean	SD	Mean	SD	Mean	SD	Over all	Fh versus S‐CAIS	Fh versus D‐CAIS	D‐CAIS versus S‐CAIS
Angular deviation (°)	6.67	3.70	2.66	1.77	3.20	2.16	< 0.001*	< 0.001*	0.002*	0.608
Global deviation at the platform (mm)	1.31	0.88	0.67	0.32	1.14	0.70	< 0.001*	< 0.001*	0.466	0.004*
Global deviation at the apex (mm)	1.75	0.90	1.00	0.39	1.23	0.58	< 0.001*	< 0.001*	0.043*	0.245
Surgical time (min)	3:15	1:21	2:55	1:12	4:27	2:04	< 0.001*	0.608	0.016*	< 0.001*

*Note*: *indicates a significant difference (*p* < 0.05).

Abbreviations: Fh, freehand; SD, standard deviation.

**FIGURE 7:**
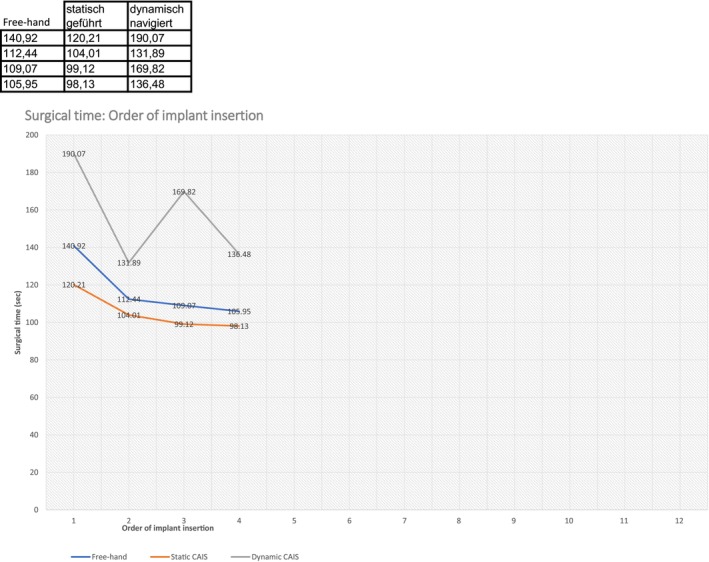
The learning curves of the freehand, static CAIS, and dynamic CAIS methods during repeated implant insertion with the same preparation technique defined by the surgical time.

With the increased number of implant placements, the surgical time continuously decreased in the FH and S‐CAIS groups (Figure 7). All surgical methods were associated with a reduced surgical time of 35, 22, and 54 s for FH, S‐CAIS, and D‐CAIS after repeated performance (Table 5). However, the insertion accuracy did not significantly differ with the increased number of placed implants in all groups.

**TABLE 5:** Average operation times of all participants separately the preparation method freehand, S‐CAIS and D‐CAIS.

	Freehand	Static CAIS	Dynamic CAIS
Region	Mean (sec)	Mean (sec)	Mean (sec)
16	140.92	120.21	190.07
11	112.44	104.01	131.89
24	109.07	99.12	169.82
26	105.95	98.13	136.48

The questionnaire results (Appendix S1) revealed that surgeons had inserted 60% of implants freehand, 35% with S‐CAIS, and 5% with D‐CAIS in the past. During this study, the participants experienced much flexibility and had to pay extra attention to FH surgery. The surgeons' self‐confidence and subjective feeling of accuracy were medium. Twelve participants chose FH as the most common technique to perform basic implant surgery.

For the S‐CAIS, scores for self‐confidence and the subjective feeling of accuracy were relatively high. Required attention and granted flexibility were evaluated to be medium. All participants demonstrated that their interest in repeating using S‐CAIS was high.

For the D‐CAIS, medium scores were found for self‐confidence, subjective feeling of accuracy, and flexibility. Considerable attention was needed while performing the surgeries, while the probability of being reused in the future was very spread in the scores.

## 4 Discussion

The initial purpose of CAIS was to improve the safety and accuracy of surgical procedures. With the evolution of technology, the workflow of digital approaches has been clarified and simplified for application in clinical practice. The incorporation of CAIS knowledge seems to be emerging in dental education. The present study compared the accuracy and learning curve of FH, S‐CAIS, and D‐CAIS approaches between experienced surgeons and dental students. The results indicated that using CAS improved implant accuracy and reduced surgical time in both population groups. Most participants showed a greater interest in using S‐CAIS in clinical practice than FH and D‐CAIS. However, the study's limitations and biases were unavoidable due to its in vitro design.

The concept of the ideal implant position involves transferring prosthetically driven planning to the surgical site with accurate placement. Therefore, various approaches have been widely studied and discussed to achieve this goal and ensure long‐term longevity. Additionally, from a patient‐centric perspective, the harm from surgery can be minimized with the help of CAIS. A study of patient‐reported outcomes found that patients experienced significantly less pain with S‐CAIS than with the FH approach after surgery. Furthermore, the FH approach was associated with the highest rate of pain medication consumption (Afrashtehfar 2021).

In some studies, no statistically significant difference between S‐CAIS and D‐CAIS was found (Wang, Wang, and Zhang 2021; Wu et al. 2020), while others showed significant differences at the apex, entry point, or angle (Jorba‐García et al. 2021; Kivovics et al. 2022; Mediavilla Guzmán et al. 2019; Spille et al. 2021). The angular deviation of the implants was statistically significantly lower in dynamic navigation when compared to S‐CAIS (Mediavilla Guzmán et al. 2019). D‐CAIS had significantly lower deviations than freehand surgery in earlier studies (Jorba‐García et al. 2019, 2021; Kivovics et al. 2022; Wang, Wang, and Zhang 2021). It was more accurate than both the S‐CAIS and FH approaches (Jorba‐García et al. 2021).

Comparable results were obtained and confirmed in the present study. Regarding the accuracy at the implant platform, S‐CAIS was significantly superior to D‐CAIS. Jorba‐García et al. (2021) found that the deviation of D‐CAIS in in vitro studies at the entry point was 0.64 mm and the angular deviation 2.01°. In clinical studies, however, deviations were significantly larger, with 1.03 mm at the entry point and an angular deviation of 3.68°. This may be explained by increased surgeon arousal or additional movement, salivation, and facial expressions.

The surgeon's experience level can also lead to different clinical outcomes. In Wu's study, experienced surgeons had significantly shorter surgical times in FH surgery and lower angular deviation in S‐CAIS compared to inexperienced surgeons (Wu et al. 2020). For D‐CAIS, a higher skill level resulted in more accurate placement in angulation but increased surgery time (Jorba‐García et al. 2019; Kunakornsawat et al. 2023). On the contrary, Pellegrino et al. found that highly skilled surgeons could reduce surgical time while maintaining accuracy. (Pellegrino et al. 2020) The evolution of the learning curve during D‐CAIS is widely unknown. Two studies have shown significant improvement in the accuracy at the entry and the apex with D‐CAIS within a series of 40 implants in both in vitro (Feng, Yao, and Yang 2022) and in vivo designs (Block et al. 2017). Despite noting a trend towards improved accuracy between the 8th and the 17th implant insertion, an unclear learning curve was observed in another study (Marques‐Guasch et al. 2022). In the present study, the learning curves regarding a continuous decrease in surgical time were only observed in the FH and S‐CAIS groups but not for D‐CAIS. Future studies should have a well‐designed protocol to evaluate the learning curve with different groups of techniques and surgeons.

The disadvantage of navigated implant surgery is that it requires a longer surgical time than the FH technique (Jorba‐García et al. 2019). A similar result was shown in the present study, which showed that D‐CAIS was much more time‐consuming than FH or S‐CAIS. In addition, planning and preparing the marker tray for the DENACAM navigation system required more time than the template design for S‐CAIS. The marker tray can be produced with a 3D printer or manually with dental silicon. In the present study, the virtual marker was positioned to the teeth‐support template in the digital planning software. The template had to be rigidly fixed to the dentition with the tracking marker during the operation. The fully digital way of producing the registration marker makes it more repeatable and accurate during registration. An alternative is to use a fabricated silicone tray for the marker fixation and combine it with CBCT and IOS for the data merging. However, manual errors from the merging process and silicone fixation may lead to extra deviations in implant surgery.

Wu et al. (2020) reported that the position of the edentulous site played a more prominent role in accuracy than the use of surgical approaches or the surgeon's experience. The in vitro experiment measured significantly higher apical deviation in the D‐CAIS group for anterior teeth. Regarding the amount and distribution of edentulous sites, a study by Kaewsiri showed no significant difference in deviation between S‐ and D‐CAIS in the region of single tooth spaces (Kaewsiri et al. 2019). Similar results were obtained in fully edentulous patients (Jaemsuwan et al. 2023) and for two implants in the same quadrant (Yimarj et al. 2020). Putra et al. (2022) assessed implant accuracy in distal free‐end and bounded edentulous spaces using S‐CAIS and identified markedly higher entry deviations in distal free‐end spaces. The present study showed similar results, with significantly higher deviations observed only after freehand implant surgery in distal free‐end spaces. Both static and dynamic computer‐assisted implant surgeries reduced these increased deviations in distal free‐end spaces.

The questionnaire results indicated that participants needed considerable concentration for FH and D‐CAIS. In contrast, statically guided implantations required moderate attention. Regarding FH implantation, surgical participants exhibited considerably more self‐assurance and adaptability than student participants. However, Ashy (2021) found no significant differences in perceived accuracy and predictability between CAIS and FH in a subjective questionnaire. These findings may be attributed to the different systems applied in each study. For surgical flexibility, compared to S‐CAIS, surgeons seem more inclined towards FH and D‐CAIS due to the ability to adjust planning according to the anatomical situation in practice. However, attention requirements were lower during S‐CAIS than during D‐CAIS and FH procedures. The selection of the approach needs to be tailored to the clinical scenario.

In the present study, the implant placement was manually inserted after preparation, as in Kühl's study (Kühl et al. 2013), which may result in more errors than mechanical insertion. However, most studies did not point out implant insertion methods (Block et al. 2017; Kaewsiri et al. 2019; Kivovics et al. 2022; Mediavilla Guzmán et al. 2019; Pellegrino et al. 2020; Schnutenhaus et al. 2021; Somogyi‐Ganss, Holmes, and Jokstad 2015; Spille et al. 2021; Wu et al. 2020; Yimarj et al. 2020) Several implant systems with different geometry designs were studied to assess the accuracy of different insertion techniques. The studies used various implant systems, namely Straumann BL (Kaewsiri et al. 2019; Spille et al. 2021; Yimarj et al. 2020), BLT (Kaewsiri et al. 2019; Schnutenhaus et al. 2021), and TL (Kaewsiri et al. 2019), as well as Camlog iSy‐Implant (Edelmann et al. 2021), BioHorizons (Mediavilla Guzmán et al. 2019), Callus Pro (Kivovics et al. 2022), Nobel Replace Tapered Groovy (Feng, Yao, and Yang 2022), and Ticare InHex standard (Jorba‐García et al. 2019). No study has focused on how different geometric designs might result in insertion deviations with CAIS approaches. Future studies should investigate this aspect. Various techniques have been used to analyze errors to evaluate planned versus placed accuracy. Some studies used IOS to produce STL files (Edelmann et al. 2021; Schnutenhaus et al. 2021), while others utilized DICOM data from CBCT scans (Block et al. 2017; Feng, Yao, and Yang 2022; Jorba‐García et al. 2019; Kaewsiri et al. 2019; Kivovics et al. 2022; Mediavilla Guzmán et al. 2019; Pellegrino et al. 2020; Somogyi‐Ganss, Holmes, and Jokstad 2015; Spille et al. 2021; Wu et al. 2020; Yimarj et al. 2020). The IOS method appears to have better accuracy (Edelmann et al. 2021) due to minimized artifacts surrounding the implants. Additionally, it is a safer approach for patients, avoiding unnecessary radiation. For the D‐CAIS, different commercial systems have been introduced to assist implant placement, such as X‐guide(Nobel Biocare AB, Sweden), Navident (ClaroNav, Toronto, ON, Canada), BrainLAB (BrainLABAG, Germany) and Dcarer (DHC‐DI242, China), which all could achieve clinically acceptable accuracy, even with the placement of zygomatic implants (Fan, Saenz‐Ravello, et al. 2023; Wang, Wang, and Zhang 2021).

The limitations of the present study includes its in vitro design and the small sample size. It is worth mentioning that using 3D‐printed models does not fully reflect the actual anatomical scenario, especially when the cortical bone is intact around the implant bed. This situation can lead to greater deviations in the apex and angular aspects. Surgeons must be aware of this limitation when placing implants using FH and CAIS.

## 5 Conclusion

The study results suggest that the accuracy of implant placement with CAIS is superior to that with FH. Highly trained surgeons may perform more accurate surgeries within less time than novice surgeons. A safety margin of 2 mm should be maintained to prevent damage to critical anatomical structures. However, additional clinical studies are required to verify the outcomes for clinical application. Furthermore, due to the limited number of implants, only an introductory phase of the learning process could be delineated. Conducting clinical studies with more implants per participant is imperative to enable complete and exhaustive investigations.

## Author Contributions

**Joscha Gabriel Werny:** investigation, funding acquisition, writing – original draft, methodology, project administration, visualization, conceptualization, data curation. **Shengchi Fan:** writing – review and editing, visualization, investigation, conceptualization, data curation. **Bilal Al‐Nawas:** writing – review and editing, visualization, data curation. **Keyvan Sagheb:** data curation, writing – review and editing. **Matthias Gielisch:** data curation, writing – review and editing, software, formal analysis. **Eik Schiegnitz:** supervision, data curation, writing – review and editing, visualization, conceptualization, investigation. **Leonardo Diaz:** conceptualization, writing – review and editing, visualization, data curation.

## Ethics Statement

Ethics approval was not required for this study.

## Conflicts of Interest

The authors declare no conflicts of interest.

## Supporting information


**Appendix S1.** Percentage of the participant’s expression of the named subjective emotions during the implant bed preparation and insertion during freehand, S‐CAIS, and D‐CAIS.

## Data Availability

The data sets used and analyzed during the current study are available from the corresponding author upon reasonable request.
