# Supplementary figures and images for: Evaluation of the Accuracy, Surgical Time, and Learning Curve of Freehand, Static, and Dynamic Computer‐Assisted Implant Surgery in an In Vitro Study
Source: Clin Oral Implants Res. 2025 Jan 21;36(5):555–65. doi: 10.1111/clr.14403 (PMC12066894; doi:10.1111/clr.14403)

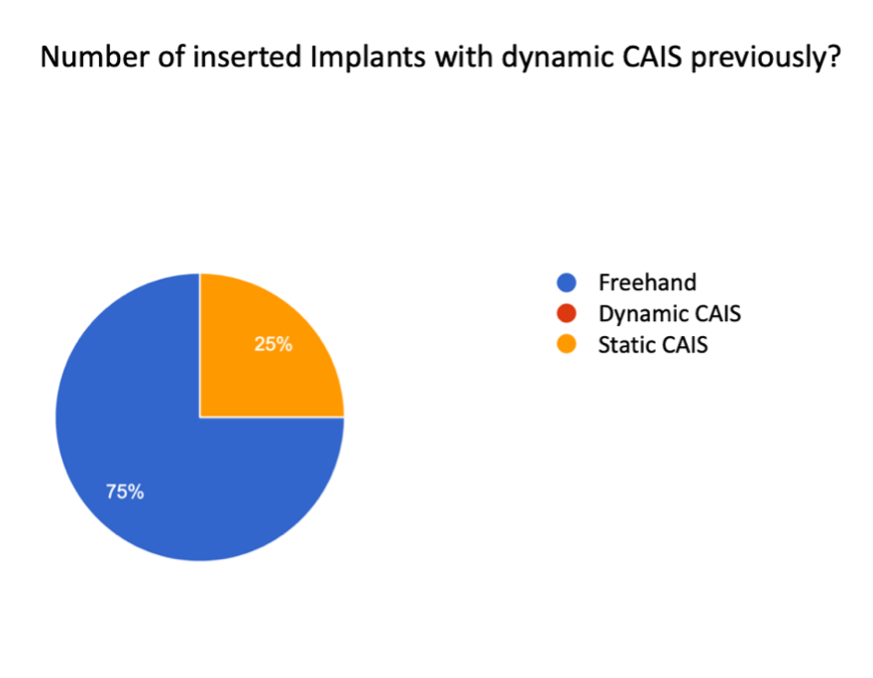

Supplement: Supplementary file 1 — Appendix S1. Percentage of the participant’s expression of the named subjective emotions during the implant bed preparation and insertion during freehand, S‐CAIS, and D‐CAIS. [file CLR-36-555-s001.zip › Appendix 1.png]

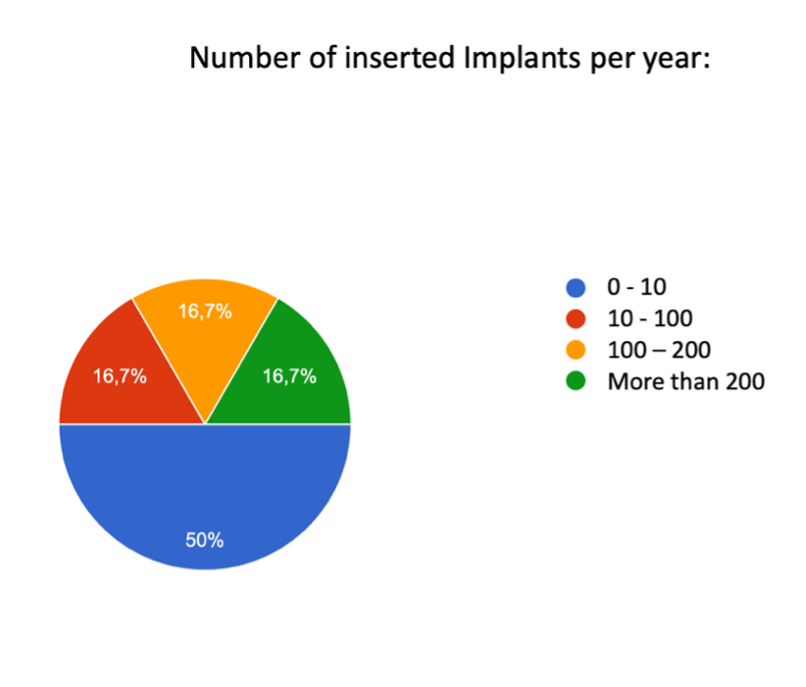

Supplement: Supplementary file 1 — Appendix S1. Percentage of the participant’s expression of the named subjective emotions during the implant bed preparation and insertion during freehand, S‐CAIS, and D‐CAIS. [file CLR-36-555-s001.zip › Appendix 2.png]

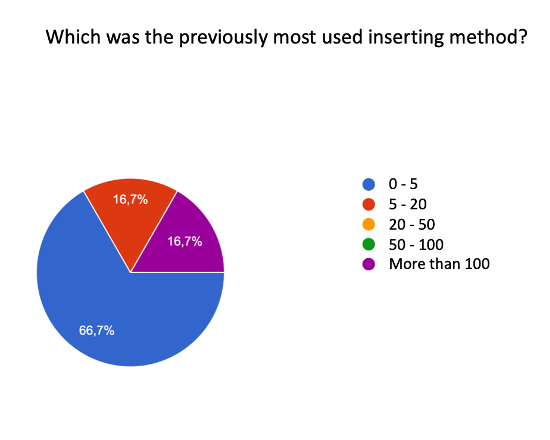

Supplement: Supplementary file 1 — Appendix S1. Percentage of the participant’s expression of the named subjective emotions during the implant bed preparation and insertion during freehand, S‐CAIS, and D‐CAIS. [file CLR-36-555-s001.zip › Appendix 3.png]

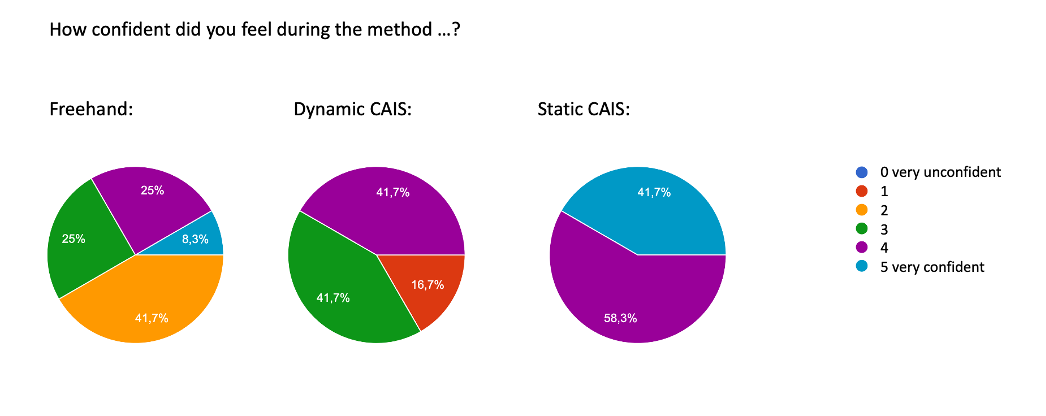

Supplement: Supplementary file 1 — Appendix S1. Percentage of the participant’s expression of the named subjective emotions during the implant bed preparation and insertion during freehand, S‐CAIS, and D‐CAIS. [file CLR-36-555-s001.zip › Appendix 4.png]

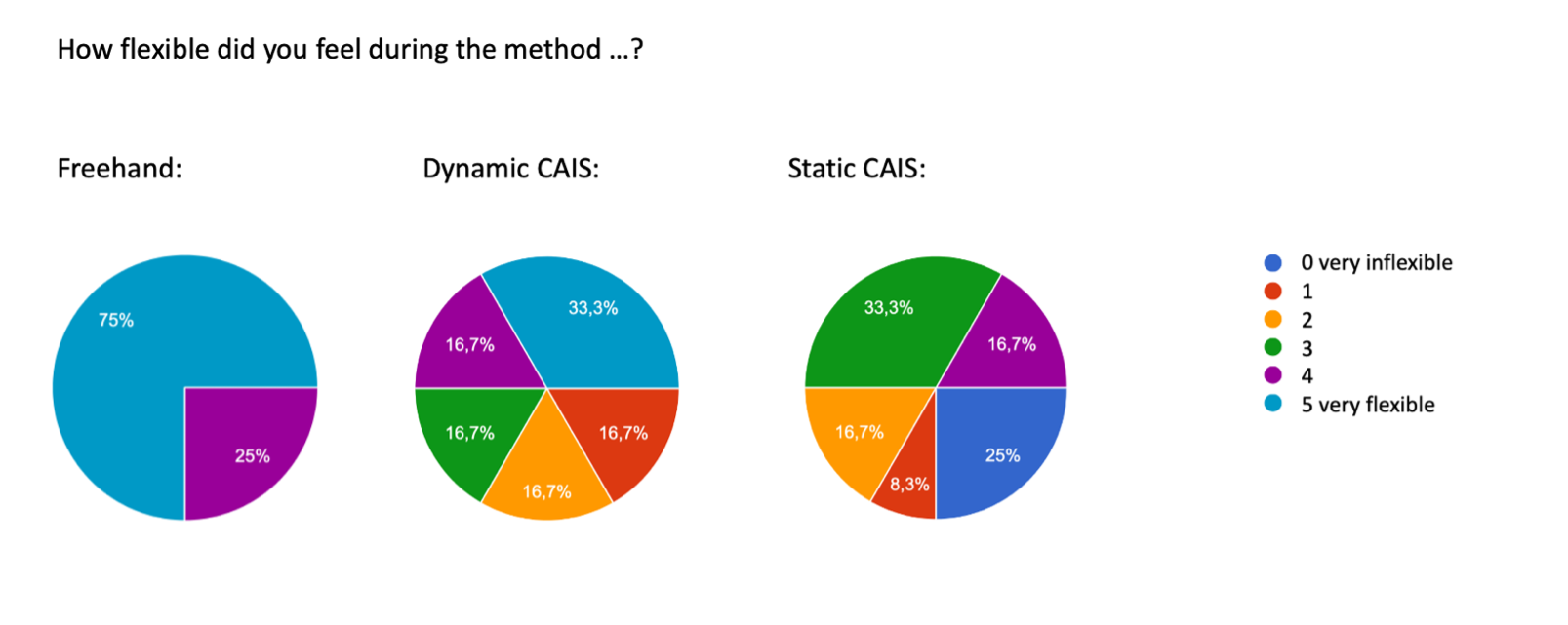

Supplement: Supplementary file 1 — Appendix S1. Percentage of the participant’s expression of the named subjective emotions during the implant bed preparation and insertion during freehand, S‐CAIS, and D‐CAIS. [file CLR-36-555-s001.zip › Appendix 5.png]

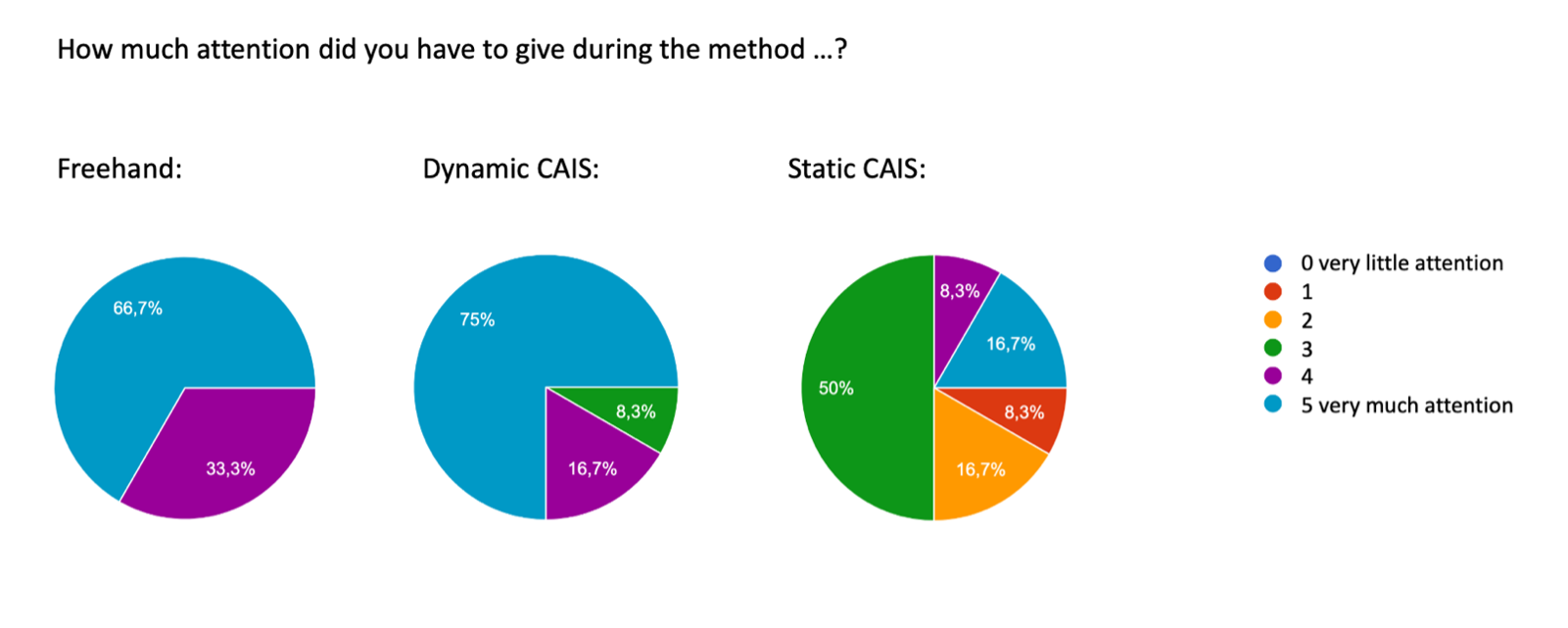

Supplement: Supplementary file 1 — Appendix S1. Percentage of the participant’s expression of the named subjective emotions during the implant bed preparation and insertion during freehand, S‐CAIS, and D‐CAIS. [file CLR-36-555-s001.zip › Appendix 6.png]

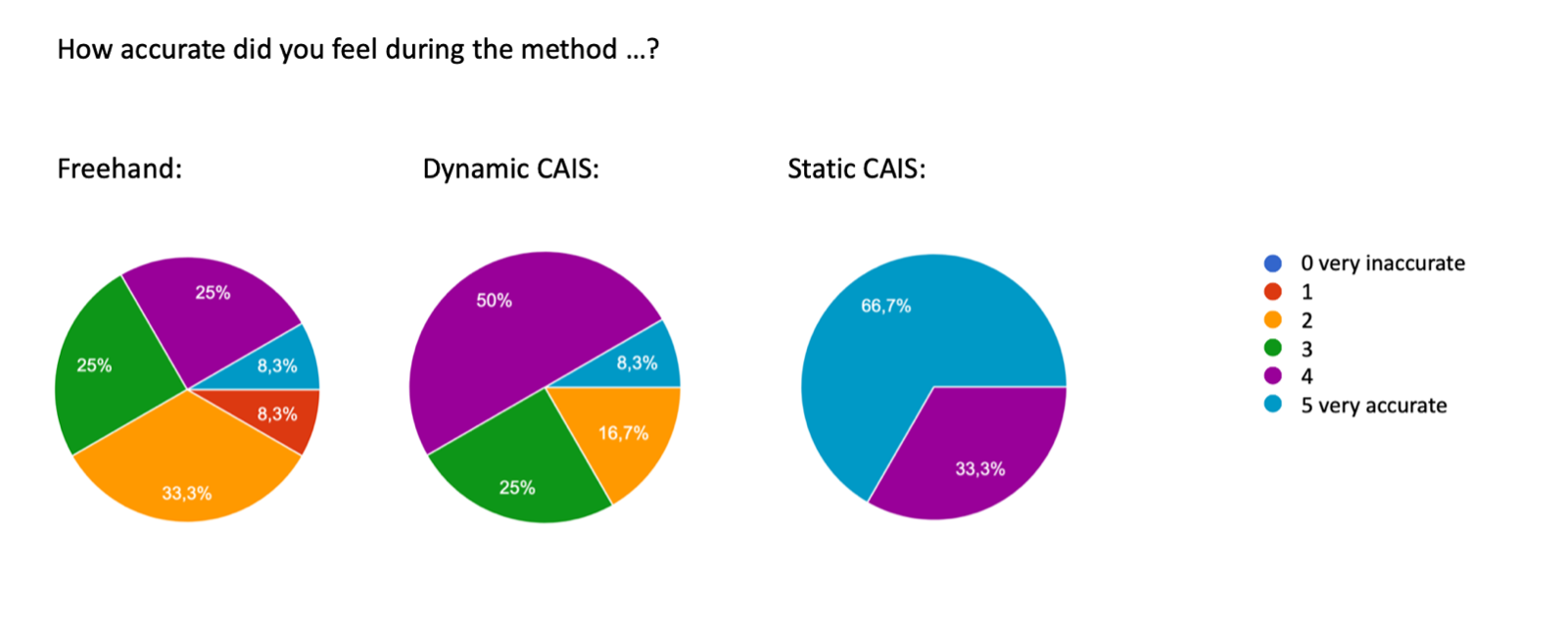

Supplement: Supplementary file 1 — Appendix S1. Percentage of the participant’s expression of the named subjective emotions during the implant bed preparation and insertion during freehand, S‐CAIS, and D‐CAIS. [file CLR-36-555-s001.zip › Appendix 7.png]

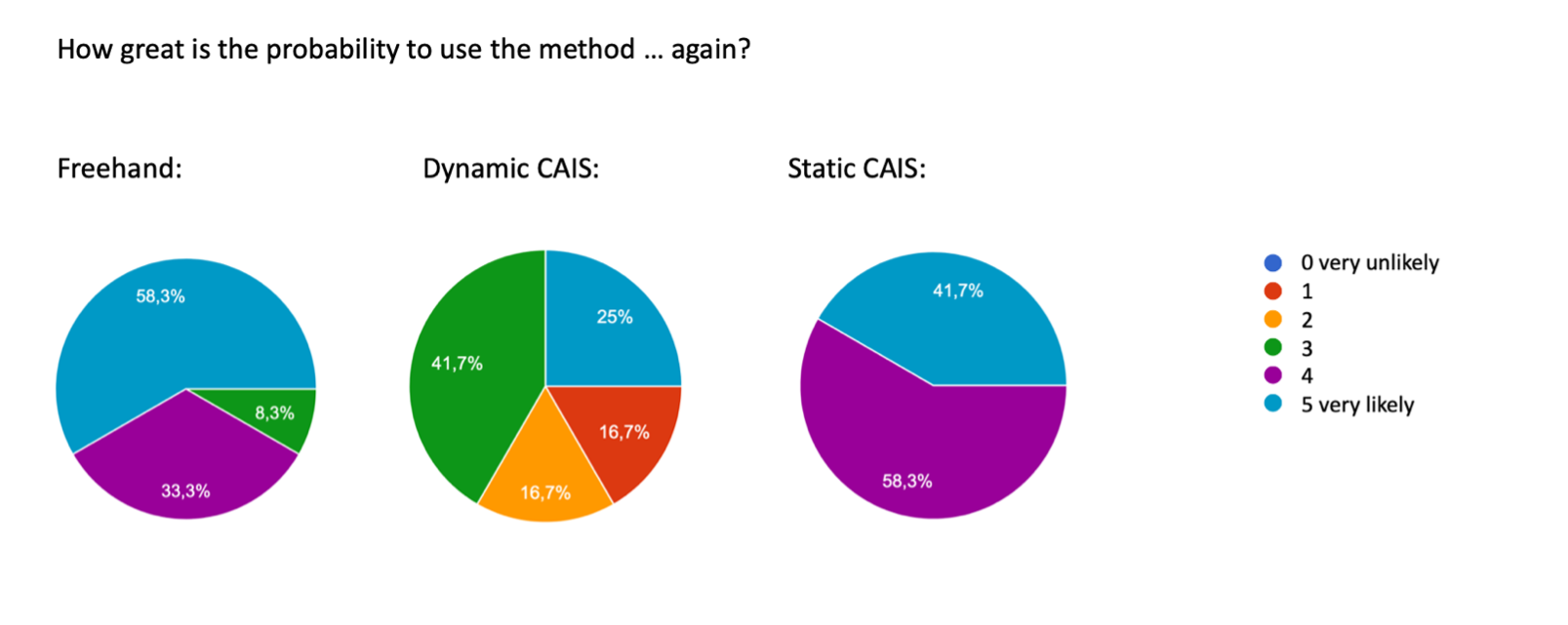

Supplement: Supplementary file 1 — Appendix S1. Percentage of the participant’s expression of the named subjective emotions during the implant bed preparation and insertion during freehand, S‐CAIS, and D‐CAIS. [file CLR-36-555-s001.zip › Appendix 8.png]
